# A novel AAV Vector for gene therapy of RPE-related retinal degenerative diseases via intravitreal delivery

**DOI:** 10.1186/s13024-024-00777-x

**Published:** 2024-11-25

**Authors:** Yajun Gong, Xianyu Huang, Tianxiang Tu, Cenfeng Chu, Chunrui Xian, Yushun Yuan, Xin Fu, Ruobi Li, Guisheng Zhong, Xiaolai Zhou

**Affiliations:** 1 State Key Laboratory of Ophthalmology, Zhongshan Ophthalmic Center, Sun Yat-Sen University, 54 Xianlie South Rd, Guangzhou, 510060 China; 2 Guangdong Provincial Key Laboratory of Ophthalmology and Visual Science, Sun Yat-Sen University, Guangzhou, 510060 China; 3 Shanghai EmayGene Biotech Co. Ltd, Shanghai, 201203 China; 4 iHuman Institute, ShanghaiTech University, Shanghai, 201210 China; 5 School of Life Science and Technology, ShanghaiTech University, Shanghai, 201210 China

**Keywords:** Retinal pigment epithelium (RPE), Adeno-associated virus (AAV), Gene therapy, Retinal degenerative diseases, Age-related macular degeneration (AMD)

**To the editor**,

Dysfunction of retinal pigment epithelium (RPE) cells leads to multiple blinding retinal degenerative diseases, including retinitis pigmentosa, age-related macular degeneration, and Stargardt disease [1]. Currently, no drug treatments are available to cure or slow the progression of these diseases, and gene therapy has been considered a promising approach. However, when delivered via intravitreal injection, commonly used vectors like AAV2 and AAV9 exhibit poor transduction rates in RPE cells. Subretinal injection, while more effective, requires sophisticated surgical skills and carries risks, such as retinal tears and detachments [2]. Therefore, developing a highly efficient RPE-specific AAV variant for intravitreal injection would be invaluable for gene therapy of RPE-related retinal degenerative diseases.

To identify such an AAV variant, we conducted a multi-round in vivo screening by intravitreal injection in mice with a randomized 9-mer library (diversity of 1.34E6), inserted between positions 587–588 of AAV2 capsid (Fig. 1A). We analyzed NGS data of collected viral genomes from three rounds of screening based on their read counts and enrichment scores, ultimately identifying 10 candidate variants (Supplemental Fig. 1A-I). Preliminary validation in mice revealed a variant with specific transduction for RPE cells, named AAV206 (Supplemental Fig. 1J). We then characterized the transduction properties of AAV206 in detail. Mice received intravitreal injections of AAV2-GFP and AAV206-GFP, and subsequent analysis of whole mounts of the RPE-choroid-sclera complex and retina showed that AAV2-GFP predominantly transduced neuroretinal cells with negligible transduction of RPE cells. In contrast, AAV206-GFP efficiently and specifically transduced RPE cells with minimal neuroretinal transduction (Fig. 1B-D). Consistent with these findings, frozen sections of the retina revealed that AAV2-GFP expression was mainly observed in retinal ganglion cell layer and inner nuclear layer, whereas AAV206-GFP specifically transduced RPE cells (Fig. 1E-F).

**Fig. 1:**
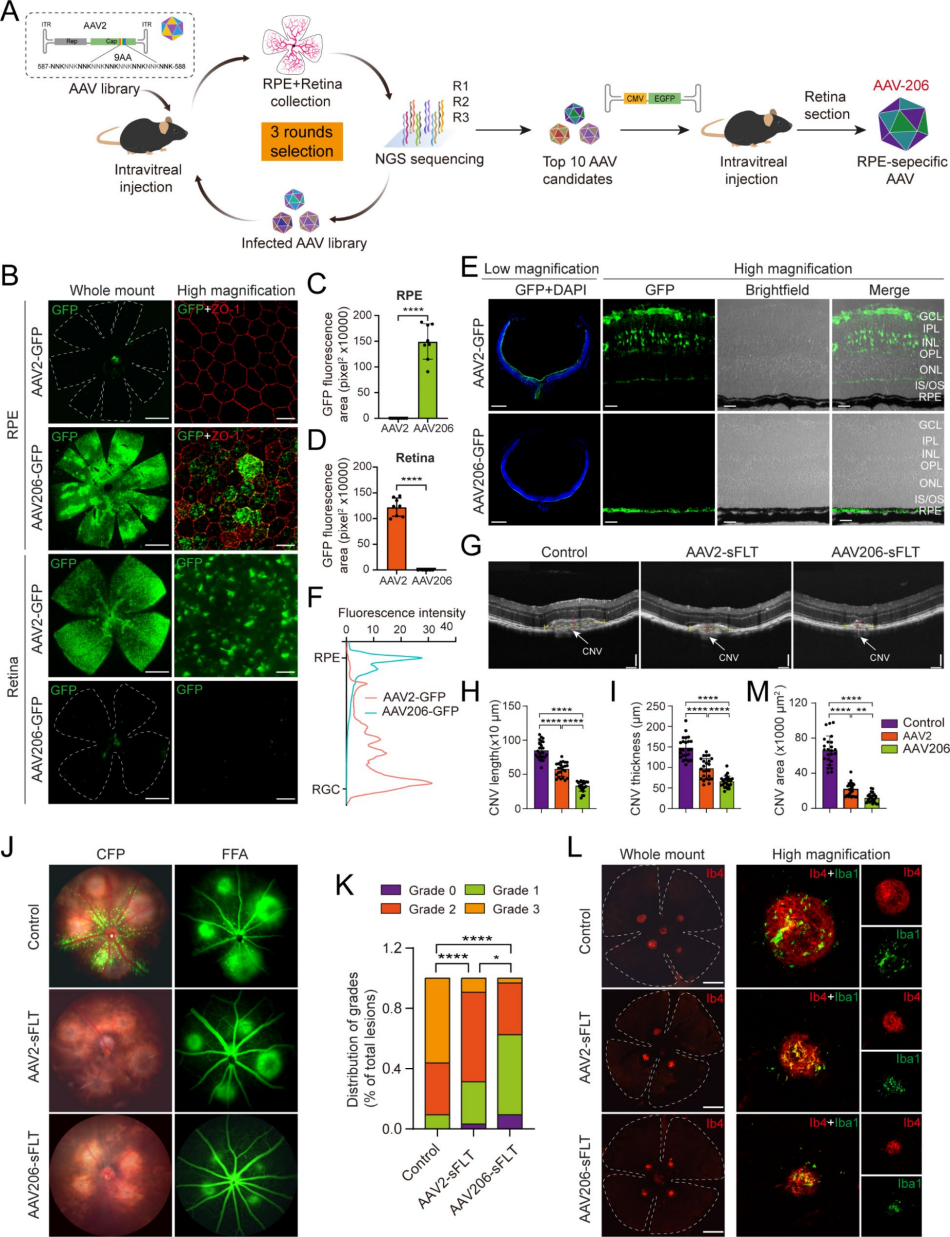
A novel RPE-specific AAV vector. **(A)** Schematic diagram of AAV vectors screened for specific targeting of RPE cells. **(B)** Whole mounts of mice retina and RPE-Choroid-Sclera after intravitreal injection of AAV2-GFP or AAV206-GFP for 14 days. GFP expression (green) indicates positively transduced cells. RPE cells are marked by ZO-1 (red). Scale bar: 1000 μm (left panel), 20 μm (right panel). **(C**,** D)** Statistical results of GFP fluorescence areas in RPE (C) and retinal (D) tissues from (B). *n* = 8 eyes, ****P < 0.0001 by Student’s *t*-test; values shown as mean ± SD. **(E)** Frozen sections of mice retina 14 days post-transduction with AAV2-GFP or AAV206-GFP. *n* = 6 eyes, Scale bar: 1000 μm (low magnification), 40 μm (high magnification). **(F)** Quantification of GFP fluorescence intensity in (E) using ImageJ. **(G)** CNV was examined in vivo by OCT after the induction of CNV for 7 days. Scale bar: 200 μm. **(H**,** I)** Measurements of CNV lesion length (yellow dotted line) and thickness (red dotted line) from OCT. *n* = 8 eyes, ****P < 0.0001 by one-way ANOVA; values shown as mean ± SD. **(J)** FFA to detect CNV lesion leakage, with representative color fundus photographs (CFP) and FFA images for each group after the induction of CNV for 7 days. **(K)** Distribution of CNV lesion grades. *P < 0.05, ****P < 0.0001 by the Kruskal-Wallis test. **(L)** RPE-choroid-sclera whole mounts stained with IB4 (red) to measure CNV areas after the induction of CNV for 7 days, with microglia marked by iba1 (green). Scale bar: 1000 μm (low magnification), 100 μm (high magnification). (**M**) Quantification of CNV areas in (L) using ImageJ. *n* = 8 eyes, **P < 0.01, ****P < 0.0001 by one-way ANOVA; values shown as mean ± SD

Additionally, we investigated the toxicity and immunogenicity of AAV206 vector. Fourteen days after intravitreal injection of AAV2 and AAV206, electroretinography results revealed no significant changes in a-wave amplitudes in either group. However, AAV2 group, but not AAV206 group, showed a significant reduction in b-wave amplitude (Supplemental Fig. 2A-C). Despite this, neither group exhibited obvious retinal degeneration (Supplemental Fig. 2D-E). To further assess the immunogenicity of both vectors, we performed RNA sequencing. The volcano plot demonstrated 898 upregulated and 915 downregulated genes in AAV206 group compared to AAV2 group (adj.P.val < 0.05, absolute logFC > 0.2) (Supplemental Fig. 2F). Gene set enrichment analysis (GSEA) indicated that the top 15 suppressed biological processes were mainly related to immune response (Supplemental Fig. 2G). Further hallmark gene-set analysis revealed that the top 15 suppressed pathways were primarily enriched in inflammatory pathways such as IL6/JAK/STAT3 and TNFα/NF-κB (Supplemental Fig. 2H). Consistently, immunofluorescence staining showed fewer activated microglia in AAV206-GFP group compared to AAV2-GFP group (Supplemental Fig. 2I-J). Together, these findings indicate that AAV206 exhibits lower retinal toxicity and immunogenicity compared to AAV2.

To explore the gene therapy efficacy of AAV206 vector in RPE-related retinal degenerative diseases via intravitreal injection, we employed a laser-induced choroidal neovascularization (CNV) mouse model for wet AMD. In this type of AMD, dysfunctional RPE cells abnormally produce VEGF, leading to pathological CNV, which can cause vascular leakage or hemorrhage in the subretinal space, ultimately resulting in retinal degeneration [3]. Given that the soluble VEGF receptor, sFlt-1, can neutralize VEGF, and that AAV2-sFLT-based gene therapy via intravitreal injection has already shown promise in treating wet AMD [4], we compared the treatment effects of intravitreal injection of AAV206-sFLT and AAV2-sFLT in wet AMD. The results from optical coherence tomography (OCT) and fluorescein fundus angiography (FFA) demonstrated that both AAV2-sFLT and AAV206-sFLT significantly reduced CNV size and leakage in vivo. However, AAV206-sFLT exhibited stronger effects on both CNV size and leakage compared to AAV2-sFLT (Fig. 1G-K). Consistently, immunofluorescence staining showed that both AAV2-sFLT and AAV206-sFLT significantly reduced CNV size, with AAV206-sFLT demonstrating superior efficacy (Fig. 1L-M). Thus, although AAV2 has demonstrated promising efficacy in CNV treatment, AAV206 may achieve better therapeutic effects with less impact on the retina.

In summary, we have identified a novel RPE-specific AAV vector, AAV206, developed through random mutations in AAV2. Delivered via intravitreal injection, AAV206 transduces RPE cells with high specificity and efficiency while exhibiting low retinal toxicity and immunogenicity compared to the conventional AAV2 vector. Moreover, we demonstrated that AAV206-sFLT, when injected intravitreally, provides superior inhibition of CNV formation compared to AAV2-sFLT in a laser-induced CNV mouse model. Whether AAV206 also shows similar efficacy in other animal models, such as non-human primates, requires further investigation. Nevertheless, these findings suggest that AAV206 vector represents a valuable tool for studying RPE biology and holds promise as an intravitreal delivery vector for treating RPE-related retinal degenerative diseases.

## Electronic supplementary material

Below is the link to the electronic supplementary material.

Supplementary Material 1

## Data Availability

All data generated or analyzed during this study are included in this published article and its supplementary information files. Bulk RNA-seq raw data has been deposited into the GEO database (accession number: GSE271661).
