# Supplementary material for: A novel AAV Vector for gene therapy of RPE-related retinal degenerative diseases via intravitreal delivery
Source: Mol Neurodegener. 2024 Nov 25;19:89. doi: 10.1186/s13024-024-00777-x (PMC11590224; doi:10.1186/s13024-024-00777-x)
Supplement: Supplementary file 1 — Supplementary Material 1 [file 13024_2024_777_MOESM1_ESM.pdf]

Supplemental Material

A Novel AAV Vector for Gene Therapy of RPE-Related Retinal Degenerative Diseases via Intravitreal Delivery

Yajun Gong[1,2*], Xianyu Huang[3*], Tianxiang Tu[1,2], Cenfeng Chu[4,5], Chunrui Xian[4,5], Yushun Yuan[3], Xin Fu[1,2], Ruobi Li[1,2], Guisheng Zhong[4,5#], Xiaolai Zhou[1,2#]

[1]State Key Laboratory of Ophthalmology, Zhongshan Ophthalmic Center, Sun Yat-Sen University, Guangzhou, 510060, China. [2]Guangdong Provincial Key Laboratory of Ophthalmology and Visual Science, Sun Yat-Sen University, Guangzhou, 510060, China. [3]Shanghai EmayGene Biotech Co. Ltd., Shanghai 201203, China. [4]iHuman Institute, ShanghaiTech University, Shanghai 201210, China. [5]School of Life Science and Technology, ShanghaiTech University, Shanghai 201210, China

[*]These authors contributed equally to this work.

[#]Senior authors for this work

Corresponding Authors

Xiaolai Zhou, M.D., Ph.D., Zhongshan Ophthalmic Center, Sun Yat-sen University, 54 Xianlie South Rd, Guangzhou 510060, China, Phone: (+8620)-8733-5146, Email: zhouxiaolai@gzzoc.com or Guisheng Zhong, Ph.D., iHuman Institute, ShanghaiTech University, Shanghai 201210, China. Email: zhongsh@shanghaitech.edu.cn

Supplemental Material Content:

Supplemental Methods

Supplemental Figures

Reference

Supplemental Methods

Animal

Six- to eight-week-old male and female C57BL/6 J mice were used in this study. The mice were housed under specific pathogen-free (SPF) conditions with unrestricted food and drinking water access in a 12 h light-dark cycle.

AAV capsid plasmid library generation

The AAV capsid plasmid was constructed as previously described **[1]**. The plasmid RC-cap2 consists of AAV2 ITRs flanking AAV2 Rep and AAV2 Cap genes. Our parental library was generated by a random 9-mer amino acid sequence insertion between N587 and R588 of the AAV2 capsid. The plasmid RC-cap2 was linearized by cutting at the BsiWI (R0553V, NEB) and SnaBI (R0130V, NEB) restriction enzyme sites. Two fragments containing a random peptide were obtained using the PCR method, with the primers shown in the figure below. Utilizing homologous recombination, two fragments

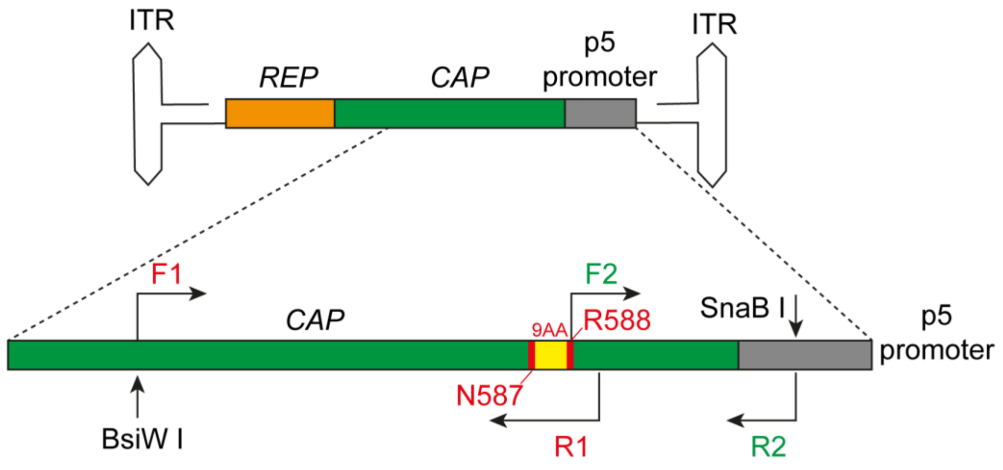

Primer sequences for library generation

Primer	Purpose	Sequence
F1	PCR1: forward	TTACTGACTCGGAGTACCAGCTCCC
R1	PCR1: reverse + Random 9-mer AA	CATCTGCGGTAGCTGCTTGTCTMNNMNNMNNMNNMNNMNN MNNMNNMNNMNNGTTGCCTCTCTGGAGGTTGGTAGATAC
F2	PCR2: forward	AGACAAGCAGCTACCGCAGATG
R2	PCR2: reverse	TTAACCCGCCATGCTACTTATCTAC

are inserted into the linearized plasmid to obtain a parental library plasmid. The parental library plasmid is then electroporated into MegaX DH10B cells (Invitrogen, C640003) for amplification.

Viral vector production and purification

The AAV vectors used in this study were produced by OBiO Technology Corp.,Ltd (Shanghai, China). The human sFlt-1 gene was subcloned into plasmid pcAAV-CMV-WPRE with slight modifications from the previously described protocol **[2]**. AAVs were packaged by transfection of HEK293T cells using polyethylenimine (PEI). For the AAV library, the capsid plasmids and pHelper plasmid in 0.22ug: 42ug ratio were co-transfected in HEK293T cells for each 150 mm dish. For recombinant AAVs, vectors were generated via a triple plasmid transfection by PEI consisting of AAV capsid plasmid (0.50 μg/mL), pHelper plasmid (0.60 μg/mL), and GOI plasmid (0.30 μg/mL). The cells were maintained at 37°C and 5% CO2 incubator until they reached approximately 80% confluence. For all of the AAV viruses, both cell lysates and supernatant were collected 96h after transfection and then AAVs were purified by iodixanol gradient ultracentrifugation **[3]**. Concentration and buffer exchange were performed against PBS containing 0.001% pluronic. The titers of AAV vector stocks were then determined based on real-time quantitative PCR.

In vivo selection

AAV libraries were injected into C57BL6/J mice at 1-month-old with intravitreal injection for 3 rounds. 6 eyes were injected in each round. For each round, the total viral genomes (vg) were 2E10, 2E10, and 3E10 vg for one eye. 72h after injection, only

retina and RPE were harvested, and DNA were extracted with the DNeasy Blood & Tissue Kit (69504, Qiagen). Subsequently, whole cell DNA from isolated retina and RPE served as a template to recover the peptide insert fragment by PCR with primers 5'-TTACTGACTCGGAGTACCAGCTCCC-3' and 5'-TTAACCCGCCATGCTACT TATCTAC-3'. The plasmid RC-cap2 was digested with restriction enzyme BsiWIand SnaBI, and then ligated with the PCR product to generate a plasmid pool for sub-library production. Whole-cell DNA from isolated Retina and RPE were amplified for next-generation sequencing (NGS) by PCR with primer 5'-GGTCATGATTACAGACGA AGAGG-3' and 5'-GGAATCTTTGCCCAGATGGG-3'. Types and numbers of viral genomes were analyzed by NGS.

NGS Data analysis

We analyzed the NGS data of collected viral genomes from three rounds, named R1, R2, and R3, by their read counts and enrichment scores. The enrichment scores of two neighboring rounds are denoted by ES1 and ES2, respectively (**Supplemental Fig. 1A**). As a control, the enrichment scores ES1 of AAV2 and PHP.eB would occupy a higher position, and the enrichment score ES2 would only drop to a lower level when proceeding to the third round of screening. This suggests that the enrichment scores in the second round are less reliable than those in the third round in reflecting the true level of enrichment of the variants. This is in line with other screening tests, where the dominant variant may be smaller in the early rounds of screening and need to be progressively enriched in more rounds of screening. ES2 for PHP.eB was 0.33, and AAV2's ES2 was already lower than 0. So in the subsequent analyses, 0.3 was used as

a threshold for the ES2 enrichment scores (**Supplemental Fig. 1B-C**).

Further, we performed cluster analysis on ES2 of AAV variants. First, we filtered the ES2 data with two filtering conditions, one is that the ES2 of AAV variants is greater than 0.3, and the other is that the number of read counts of AAV variants in R2 and R3 is greater than or equal to 5. After filtering, we obtained a total of 169 variants, constructed 373 connections by edit distance 1AA, and obtained four amino acid family maps, Family A-D. The core variants of each family as variant candidates, named as 201, 203, 205 and 207 (**Supplemental Fig. 1 D-G**). However, with the exception of Family D, the core variants of the other three families were not the ones with the highest ES values; therefore, we additionally chose the variants with the highest ES2 of each family to be the candidates, named 202, 204 and 206 (**Supplemental Fig. 1H**). In addition, for the variants that met the filtering criteria but did not form clusters, we also selected three variants with higher ES2 for validation, names as 208, 209, 210 (**Supplemental Fig. 1I**). Therefore, through analysis and screening, we obtained a total of 10 AAV variant candidates.

Intravitreal injections of AAV vector

The mice were intraperitoneally anesthetized with pentobarbital (50mg/kg) and their pupils were dilated using compound tropicamide eye drops. Two microliters ($1.11x10^{12}$ v.g./ml) of AAV was gently injected into left eye of each mouse using a 33-g Hamilton syringe. The Mice were resuscitated on an electric heating blanket and then returned to their cages.

Mouse Model of Choroidal Neovascularization (CNV)

Seven days after intravitreal injection of AAV vectors, Six- to eight-week-old mice were intraperitoneally anesthetized with pentobarbital (50mg/kg). Then, compound tropicamide eye drops and phenylephrine eye drops were mixed and administered to dilute the pupils. Using a contact lens, four laser burns were created around the optic disc at a distance of 2 to 3 papillary diameters with an ophthalmic therapy laser (IRIDEX 810 nm, Mountain View, CA, USA) through a slit lamp delivery system. The laser parameters were set to 200 mW power, 50 ms duration, and a 100 μm spot size. The model is considered successful only if bubbles appear after laser photocoagulation.

Flat mount and Immunostaining

After euthanizing the mice with an overdose of pentobarbital, the eyeballs were enucleated and fixed in 4% paraformaldehyde for 30 minutes. The neural retina or the RPE-choroid-sclera complex was dissected and washed 3 times with PBS. Then tissues were permeabilized and blocked 10% goat serum containing 0.5% Triton x-100 (Sigma) for 1 h at room temperature. Depending on the experimental conditions, the tissues were incubated with the primary antibody: ZO-1 monoclonal antibody (Invitrogen, 33-9100), GS-IB4, Alexa Fluor™ 594 (Invitrogen, I21413), Iba1 antibody (019-19741, Wako, Japan). After overnight incubation at 4°C, the samples were washed 3 times with PBS and incubated with the appropriate fluorescent secondary antibodies. Finally, the tissues were mounted with an anti-fade mounting medium (H-1900, Vector laboratories, USA) and imaged using a confocal microscope (LSM 880, Carl Zeiss, Germany). The area of CNV and GFP fluorescence were measured using ImageJ software (version 1.54d, NIH, USA).

Frozen section and immunostaining

After euthanizing the mice, the eyeballs were gently enucleated and fixed in 4% paraformaldehyde for 30 minutes seven days post-laser photocoagulation. Then, the eyeballs were washed 3 times with PBS. After three washes with PBS, the cornea and lens were removed. The eye cups were then dehydrated through a sucrose gradient and dehydrated overnight at 4°C. The eye cups were embedded in Tissue-Tek OCT compound (Sakura Finetek, Torrance, USA) and cryosectioned at 10 μm thickness. Depending on the experimental conditions, sections were permeabilized and blocked with 10% goat serum containing 0.5% Triton x-100 (Sigma) for 1 h at room temperature, incubated with anti-Iba1 antibody (019-19741, Wako, Japan) overnight at 4°C, washed three times with PBS, and then incubated with the appropriate secondary antibody. Then, the cell nuclei were counterstained using 4,6-diamidino-2-phenylindole (DAPI) for 10 min. Finally, the tissues were mounted with a mounting medium and imaged using a confocal microscope (LSM 880, Carl Zeiss, Germany). The fluorescence intensity was analyzed by ImageJ software.

H&E staining

Fourteen days after intravitreal injection of AAV vectors, mouse eyeballs were collected. The eyeballs were embedded in paraffin and sectioned, followed by hematoxylin and eosin (HE) staining. Sections were then visualized and imaged using an optical microscope digital system (ECLIPSE Ni-U, Nikon, Japan). Retinal thickness was measured using ImageJ software.

Electroretinography (ERG)

ERG was performed 14 days after intravitreal injection of AAV vectors. Before examination, mice were dark-adapted for 12 hours. Mice were fully anesthetized with pentobarbital. Then, compound tropicamide and phenylephrine hydrochloride were applied to the eyes to dilate the pupils. Retinal scotopic and photopic electrophysiological responses to a series of light intensities were recorded using the Celeris (Diagnosys, USA).

Optical coherence tomography (OCT)

Seven days after laser photocoagulation, mice were fully anesthetized. Then, compound tropicamide and phenylephrine hydrochloride were applied to the eyes to dilate the pupils. The cornea was moistened with 2% carboxymethylcellulose sodium eye drops. CNV was examined using a comprehensive retinal imaging system (Phoenix, MicronIV, USA). OCT was performed in B-scan mode to scan the area with the largest CNV lesion. The length and width of CNV were measured using ImageJ.

Fundus photography and Fundus fluorescein angiography (FFA)

FFA was used to evaluate the leakage of CNV seven days after laser photocoagulation. After OCT examination, fundus photographs of the mice were taken using the Phoenix system (MicronIV, USA). Subsequently, intraperitoneal injection of 2% sodium fluorescein (0.1 ml/kg BW) (Alcon, Japan) was administered, and early phase (1 minute) and late phase (5 minutes) fluorescence angiography images were immediately captured using the Phoenix system. The leakage of CNV lesions was graded according to a previously established scale **[4, 5]**. Briefly, Laser spots were graded and scored as follows: Grade 0 received zero points, Grade 1 received 1 point, Grade 2 received 2

points, and Grade 3 received 3 points. The total score for each group was calculated by summing the scores of all laser spots within the group.

Bulk RNA sequencing

To assess retinal changes, mice aged 6-8 weeks were divided into three groups, with five mice per group. The groups received intravitreal injections of PBS, AAV2-GFP, and AAV206-GFP, respectively. Fourteen days after intravitreal injection, retinas were harvested for bulk RNA sequencing. Total RNA was extracted from retina using Trizol reagent (15596018CN, Invitrogen, USA), following the manufacturer's protocol. RNA quantity and integrity were assessed using Qubit® RNA Assay Kit in Qubit® 2.0 Flurometer (Life Technologies, CA, USA) and the RNA Nano 6000 Assay Kit of the Bioanalyzer 2100 system (Agilent Technologies, CA, USA). Libraries for RNA sequencing were prepared using NEBNext® Ultra™ RNA Library Prep Kit for Illumina® (NEB, USA), and sequencing was performed on an Illumina NovaSeq 6000 platform to generate paired-end reads of 150 bp. Data preprocessing included quality control, read mapping to the reference genome, and quantification of gene expression levels using FastQC v0.12.1, STAR v2.7.10b, and RSEM v1.3.1, respectively. Differential expression analysis of two groups was performed using the DESeq2 R package (1.40.2). Differentially expressed genes (DEGs) were defined as those with an adjusted p-value < 0.05 and an absolute log fold change (logFC) > 0.2. Gene Set Enrichment Analysis (GSEA) was conducted using clusterProfiler v.4.8.2.

Data availability

Bulk RNA-seq raw data has been deposited into the GEO database (accession number:

GSE271661).

Statistical analysis

All data are expressed as the means ± SD. Data from three groups were compared using a one-way ANOVA followed by Dunnett's test for multiple comparisons. Two-tailed Student's t-test was used to compare the means between the two groups. Since CNV leakage scores are ordinal data, the Kruskal-Wallis test was used for statistical analysis. All analyses were performed using GraphPad Prism 10.0 (GraphPad Prism Software, Boston, MA, USA) and SPSS 27.0 software (IBM Corporation, Armonk, NY, USA).

Supplemental Figures

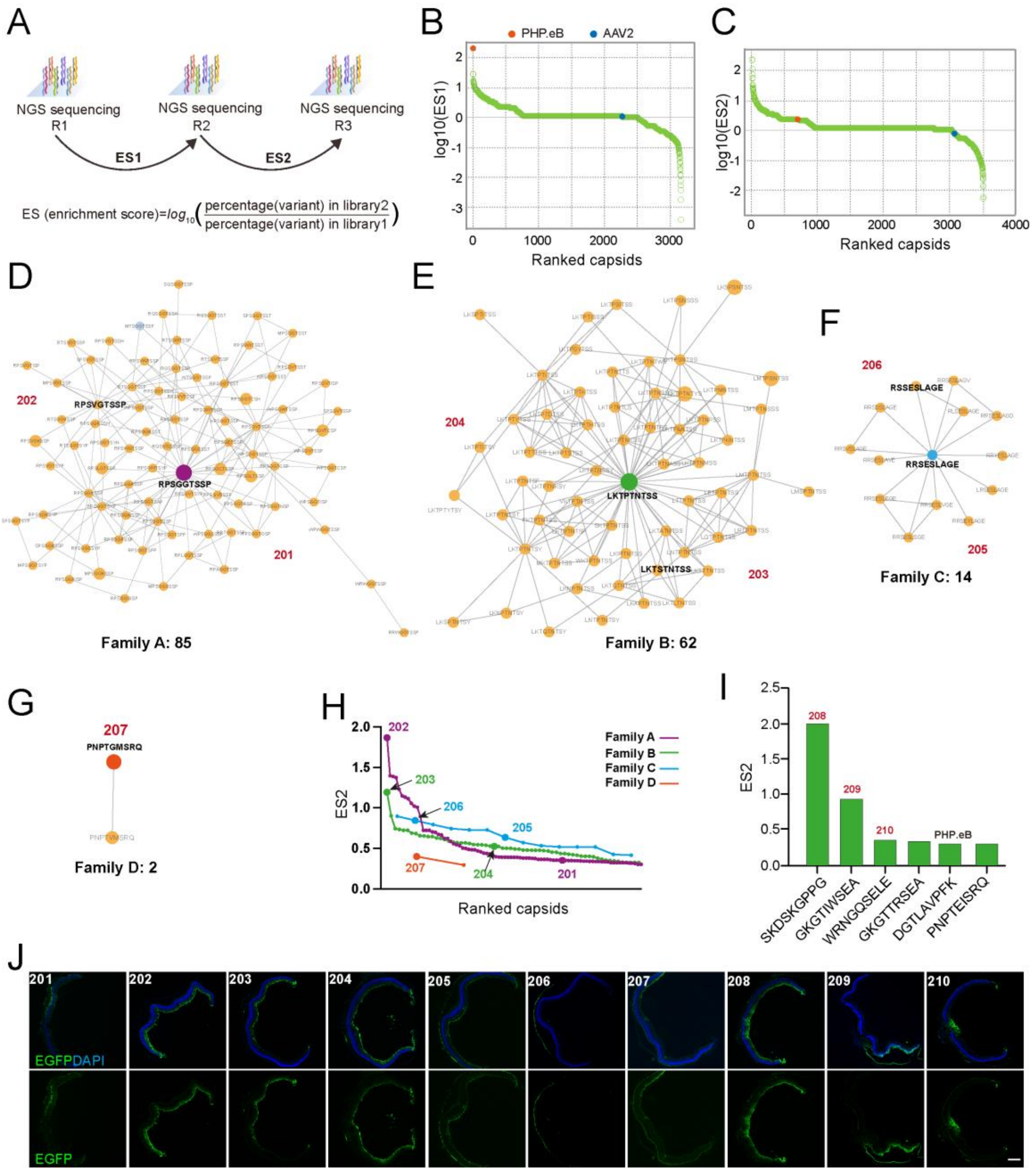

Supplementary Figure. 1. ***In vivo*** **screening of AAV2-based capsid variants specific for RPE transduction through intravitreal injection. (A)** Schematic diagram of enrichment fraction and formula. **(B-C)** Distributions of variants recovered from round 2 and round 3 are shown with capsid libraries sorted by decreasing order of the enrichment score. The enrichment score of AAV2 and PHP.eB are mapped on this plot.

(D-G) Clustering analysis of 4 typical families from positively enriched variants after filtration. Each circle represents an AAV variant, and short links indicate a difference of one amino acid between two variants. The colored circles indicated the core variants of the family. **(H)** Enrichment Score analysis of AAV variants from 4 families. The enlarged circles represent the selected AAV candidates in each family. **(I)** Enrichment Score analysis of AAV variants except for 4 families. **(J)** Representative confocal images of retina cross-section samples injected with 10 AAV candidates. ssAAV-CMV-EGFP was packaged into the indicated capsid and separately injected into at least three adult mice per candidate AAV variants by intravitreal injection. Tissues were collected 21 days after injection. Blue, DAPI; green, EGFP. Scale bar 200μm.

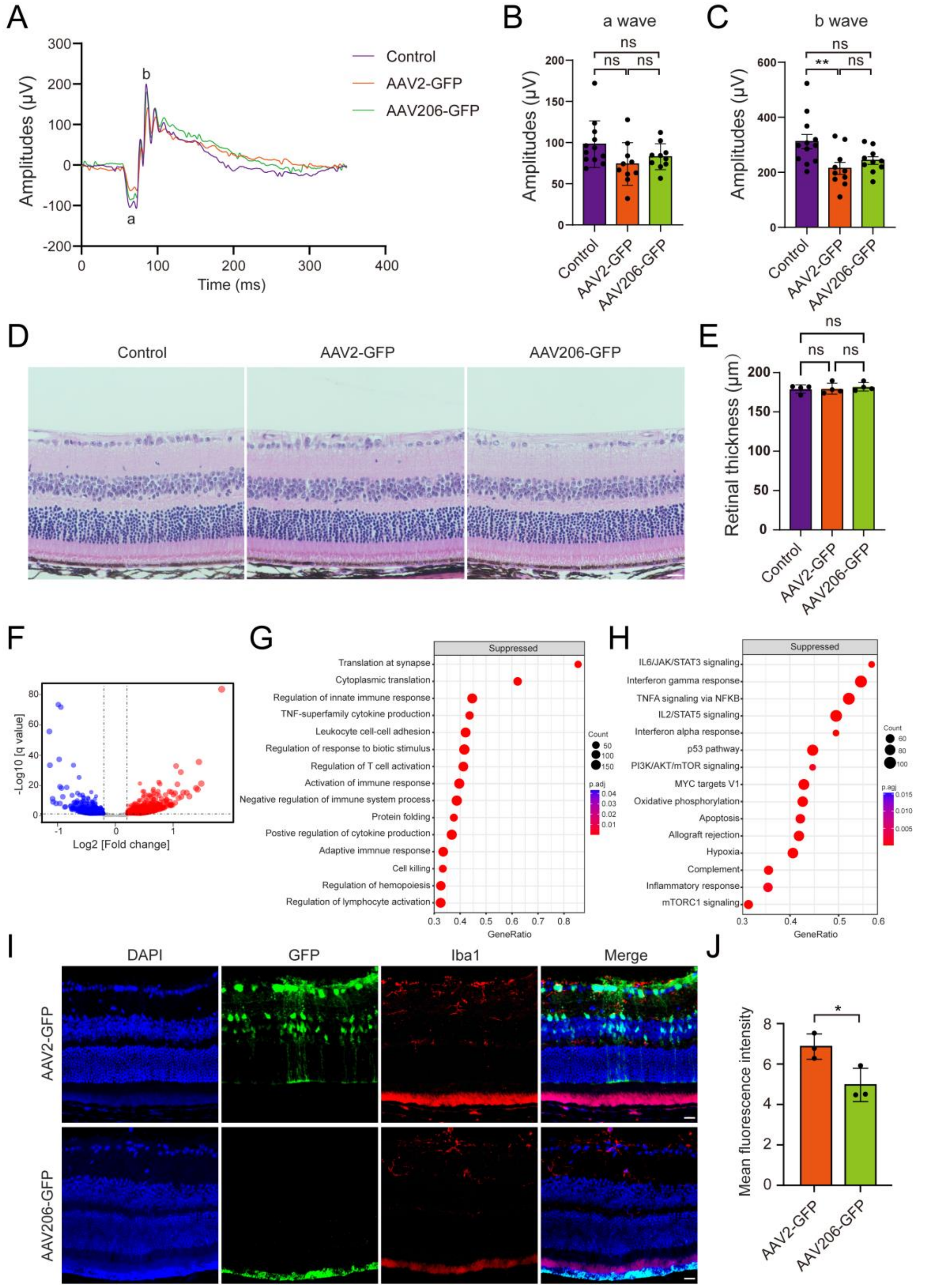

Supplemental Figure 2. Evaluation of the toxicity and immunogenicity of AAV206 vector after intravitreal injection. (A) Changes in flash scotopic ERG under the 1.0 cd.s/m² stimulus condition after intravitreal injection of PBS (Control), AAV2-GFP and

AAV206-GFP in mice, respectively. **(B)** and **(C)** represent statistical results of ERG a- and b-waves, respectively. n = 10 eyes, **p < 0.01 by one-way ANOVA. Data presents as Mean ± SD. **(D-E)** Representative pictures and statistical bar graphs of HE staining of retinal pathological sections. Scale bar 20μm, n = 4 eyes, ns by one-way ANOVA. **(F)** A volcano plot of differentially expressed genes from retinal RNA-seq data, red indicates upregulated genes and blue indicates downregulated genes. adj.p.val < 0.05 and absolute logFC > 0.2. **(G)** Top 15 suppressed pathways of gene set enrichment analysis (GSEA) with the biological process (BP) gene set. **(H)** Top 15 suppressed pathways of GSEA with the hallmark gene set. **(I)** Representative pictures of immunofluorescence staining in frozen sections of mouse retina. GFP (green) indicates positively transduced cell, microglia were marked by iba1 (red), and nuclei are counterstained with DAPI. Scale bar 20μm. **(J)** Quantification of mean fluorescence intensity in $Iba1^{+}$ microglia, n = 3 eyes, *p <0.05 by Student's *t* test.

Reference

1. Chan KY, Jang MJ, Yoo BB, Greenbaum A, Ravi N, Wu WL, Sánchez-Guardado L, Lois C, Mazmanian SK, Deverman BE, Gradinaru V. Engineered AAVs for efficient noninvasive gene delivery to the central and peripheral nervous systems. Nat Neurosci. 2017;20(8):1172-1179.
2. Lai YK, Shen WY, Brankov M, Lai CM, Constable IJ, Rakoczy PE. Potential long-term inhibition of ocular neovascularisation by recombinant adeno-associated virus-mediated secretion gene therapy. Gene Ther. 2002 Jun;9(12):804-13.
3. Chen YH, Keiser MS, Davidson BL. Adeno-Associated Virus Production, Purification, and Titering. Curr Protoc Mouse Biol. 2018;8(4): e56.
4. Krzystolik MG, Afshari MA, Adamis AP, Gaudreault J, Gragoudas ES, Michaud NA, Li W, Connolly E, O'Neill CA, Miller JW. Prevention of experimental choroidal neovascularization with intravitreal anti-vascular endothelial growth factor antibody fragment. Arch Ophthalmol, 2002, 120(3): 338-346.
5. Lai K, Li Y, Gong Y, Li L, Huang C, Xu F, Zhong X, Jin C. Triptolide-nanoliposome-APRPG, a novel sustained-release drug delivery system targeting vascular endothelial cells, enhances the inhibitory effects of triptolide on laser-induced choroidal neovascularization. Biomed Pharmacother. 2020;131: 110737.
